# Layer-specific strain in patients with heart failure using cardiovascular magnetic resonance: not all layers are the same

**DOI:** 10.1186/s12968-020-00680-6

**Published:** 2020-12-03

**Authors:** Lingyu Xu, Joseph J. Pagano, Mark J. Haykowksy, Justin A. Ezekowitz, Gavin Y. Oudit, Yoko Mikami, Andrew Howarth, James A. White, Jason R. B. Dyck, Todd Anderson, D. Ian Paterson, Richard B. Thompson, for the AB HEART Investigators, Kelly Narine, Jennifer Beck, Lisa Tanguay, Beverly Armstrong, Marleen Irwin

**Affiliations:** 1 Department of Biomedical Engineering, University of Alberta, 1098 Research Transition Facility, 8308-114 Street, Edmonton, Alberta, T6G 2V2 Canada; 2 Division of Cardiology, University of Alberta, Edmonton, Canada; 3 College of Nursing and Health Innovation, The University of Texas Arlington, Arlington, USA; 4 Canadian VIGOUR Centre, University of Alberta, Edmonton, Canada; 5 Stephenson Cardiac Imaging Centre, Libin Cardiovascular Institute of Alberta, Calgary, Canada; 6 Departments of Cardiac Sciences and Radiology, University of Calgary, Calgary, Canada; 7 Department of Pediatrics, University of Alberta, Edmonton, Canada; 8 Cumming School of Medicine, University of Calgary, Calgary, Canada

**Keywords:** Layer-specific global longitudinal strain, Cardiovascular magnetic resonance imaging, Feature tracking, Heart failure, Prognosis

## Abstract

**Background:**

Global longitudinal strain (GLS), most commonly measured at the endocardium, has been shown to be superior to left ventricular (LV) ejection fraction (LVEF) for the identification of systolic dysfunction and prediction of outcomes in heart failure (HF). We hypothesized that strains measured at different myocardial layers (endocardium = ENDO, epicardium = EPI, average = AVE) will have distinct diagnostic and predictive performance for patients with HF.

**Methods:**

Layer-specific GLS, layer-specific global circumferential strain (GCS) and global radial strain (GRS) were evaluated by cardiovascular magnetic resonance imaging (CMR) feature tracking in the Alberta HEART study. A total of 453 subjects consisted of healthy controls (controls, n = 77), at-risk for HF (at-risk, n = 143), HF with preserved ejection fraction (HFpEF, n = 87), HF with mid-range ejection fraction (HFmrEF, n = 88) and HF with reduced ejection fraction (HFrEF, n = 58). For outcomes analysis, CMR-derived imaging parameters were adjusted with a base model that included age and N-terminal prohormone of b-type natriuretic peptide (NT-proBNP) to test their independent association with 5-year all-cause mortality.

**Results:**

GLS_EPI distinguished all groups with preserved LVEF (controls − 16.5 ± 2.4% vs. at-risk − 15.5 ± 2.7% vs. HFpEF − 14.1 ± 3.0%, p < 0.001) while GLS_ENDO and all GCS (all layers) were similar among these groups. GRS was reduced in HFpEF (41.1 ± 13.8% versus 48.9 ± 10.7% in controls, p < 0.001) and the difference between GLS_EPI and GLS_ENDO were significantly larger in HFpEF as compared to controls. Within the preserved LVEF groups, reduced GRS and GLS_EPI were significantly associated with increased LV mass (LVM) and LVM/LV end-diastolic volume EDV (concentricity). In multivariable analysis, only GLS_AVE and GRS predicted 5-year all-cause mortality (all ps < 0.05), with the strongest association with 5-year all-cause mortality by Akaike Information Criterion analysis and significant incremental value for outcomes prediction beyond LVEF or GLS_ENDO by the likelihood ratio test.

**Conclusion:**

Global strains measured on endocardium, epicardium or averaged across the wall thickness are not equivalent for the identification of systolic dysfunction or outcomes prediction in HF. The endocardium-specific strains were shown to have poorest all-around performance. GLS_AVE and GRS were the only CMR parameters to be significantly associated with 5-year all-cause mortality in multivariable analysis. GLS_EPI and GRS, as well as the difference in endocardial and epicardial strains, were sensitive to systolic dysfunction among HF patients with normal LVEF (> 55%), in whom lower strains were associated with increased concentricity.

## Introduction

Heart failure (HF) is a complex clinical syndrome with a wide array of characteristic cardiac structural and functional abnormalities [1]. Patients are typically categorized according to left ventricular (LV) ejection fraction (LVEF), with HF and reduced ejection fraction defined as LVEF < 40% (HFrEF), HF with mid-range LVEF in the span of 40–49% (HFmrEF) and those with HF with preserved LVEF ≥ 50% (HFpEF) [2, 3]. However, LVEF has an inconsistent relationship with outcomes [4, 5] and does not distinguish those with preserved LVEF from those without cardiac disease [6]. Global longitudinal strain (GLS) provides an alternate measure of systolic dysfunction that has been shown to be superior to LVEF in distinguishing HF patients from healthy subjects [6] and predicting adverse outcomes in acute HF [7].

However, myocardial structure and function are heterogeneous, with layer-specific fiber orientations ranging from largely circumferential at the mid-wall to more oblique at the endocardium (ENDO) and epicardium (EPI) [8]. There is a gradient in myocardial strain across the wall, with decreasing values from ENDO to EPI [9, 10] which makes reported values dependent on measurement layer, with endocardial values being most commonly reported [6, 7, 11, 12]. The potential added value of layer-specific strain has been illustrated in various cardiovascular diseases. In those with suspected coronary artery disease, ENDO strains were shown to be superior to EPI strains for diagnosis [13]. Other studies of ischemic heart disease have shown the superior performance of ENDO strain for the prediction of outcomes [14, 15]. Conversely, EPI longitudinal strain has been shown to provide incremental prognostic information for acute coronary syndrome [16] and hypertension [17], and improved diagnostic performance for myocarditis [18]. EPI, mid-wall and endocardial GLS were shown to have similar predictive performance in population-based cohort [19]. Layer-specific strains were recently reported in a small HF cohort [10].

The aims of the current study were to compare the utility of global strains assessed at different layers of the myocardium, measured with cardiovascular magnetic resonance imaging (CMR) feature-tracking, to characterize layer-dependence, to detect systolic dysfunction in HFpEF and to predict outcomes in all HF patients, regardless of LVEF.

## Methods

### Study population

The study was approved by the University of Alberta and University of Calgary Health Research Ethics Boards and all study participants provided written informed consent. We excluded those unable to provide informed consent or with a contraindication to CMR. Patient recruitment and testing has previously been reported [20]. Briefly, we recruited patients with chronic HF and those at-risk for HF from ambulatory clinics and all subjects underwent comprehensive phenotyping that included a detailed history and physical examination, serum biomarkers and a multi-parametric CMR exam. Individuals at-risk for HF had a history of coronary artery disease, diabetes mellitus, hypertension, atrial fibrillation, and/or obesity without a diagnosis of HF (American Heart Association (AHA)/American College of Cardiology (ACC) class A and B) [20]. HF patients (AHA/ACC Class C), were sub-grouped into those with preserved (HFpEF, LVEF ≥ 55%) [21, 22], midrange (HFmrEF, 40% ≤ LVEF < 55%) or reduced ejection fraction (HFrEF, LVEF < 40%) [2, 3]. Healthy controls were also recruited and underwent identical testing.

### CMR protocol

All subjects underwent a CMR examination on 1.5 T CMR scanners (Siemens Sonata or Avanto, Siemens Healthineers, Erlangen, Germany) at the University of Alberta or University of Calgary sites, respectively. To quantify cardiac structure and function, the CMR exam included standard balanced steady state free precession (bSSFP) cine imaging, with 10–14 short axis slices covering the entire ventricle, as well as two-chamber, three-chamber and four-chamber long axis views. Typical acquisition parameters: Repetition time/echo time (TR/TE) 2.8/1.4 ms, 50–70 degree flip angle, 8 mm slice thickness with a 2 mm gap for short axis slices, 256 × 192 matrix, 380 × 285 mm field of view, 10 views per segment with 25 or 30 reconstructed cardiac phases over the cardiac cycle. All cardiac images were acquired with electrocardiographic gating within an 8–12 s breath-hold per slice.

### Image analysis

LV and right ventricular (RV) end-diastolic volume (LVEDV, RVEDV) and end-systolic volume (LVESV, RVESV), ejection fraction (LVEF, RVEF) and left ventricular mass (LVM) were measured using Syngo Argus, (Siemens Healthineers) or CVI42 (Circle Cardiovascular Imaging, Calgary, Canada). Both methods used only short axis slices for volume analysis and papillary muscle was included in the chamber volume. Volumes and LVM were normalized to body surface area (BSA), calculated using idealized weight for the given subject height [23]. Relative wall thickness (RWT) was calculated from short-axis slices as average end-diastolic wall thickness divided by average LV end-diastolic diameter from two short-axis slices (mid and mid-basal slice locations). LVM/LVEDV (concentricity) was also calculated.

**Fig. 1:**
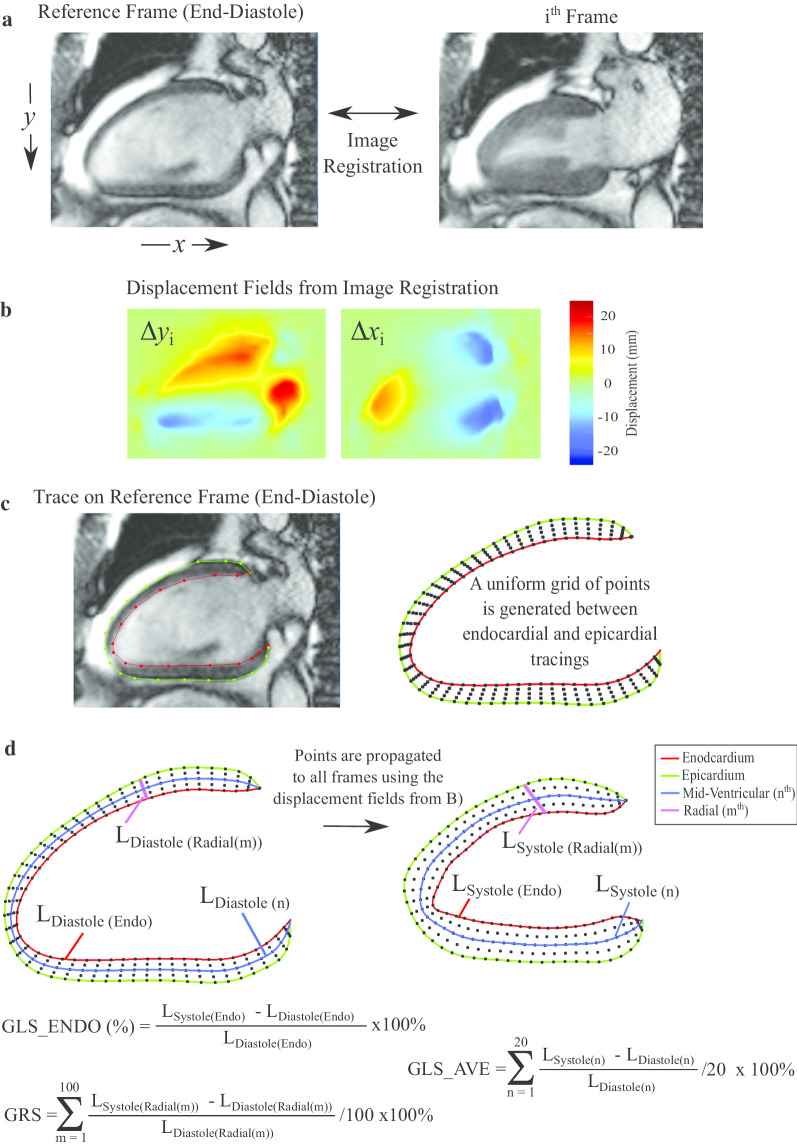
Strain Method. **a** Registration of cine images is used to calculate pixel-wise displacement fields between each image frame and the reference frames. **b** The process of registration yields displacement images (∆*x*_*i*_, ∆*y*_*i*_) that contain the translation of each pixel from the reference frame to the target ith image frame. **c** The user specifies endocardial (red) and epicardial (green) points on the end-diastolic frame. A uniform grid of points is generated at end-diastole consisting of 20 equally spaced contours between the user-specified endocardial and epicardial tracings from, with 100 equally spaced points along each contour. **d** Each point, or equivalently, each series of points in a contour, is propagated to all other cardiac phases using the previously calculated displacement fields. Four sample contours are shown. Strain for each contour is calculated as the fraction change in the contour length to end-systole. For regions that contain multiple contours, as shown here for GLS_AVE and GRS, average strains are calculated as the average of the strains from the individual contours. GLS_AVE, average global longitudinal strain; GRS, global radial strain

**Fig. 2:**
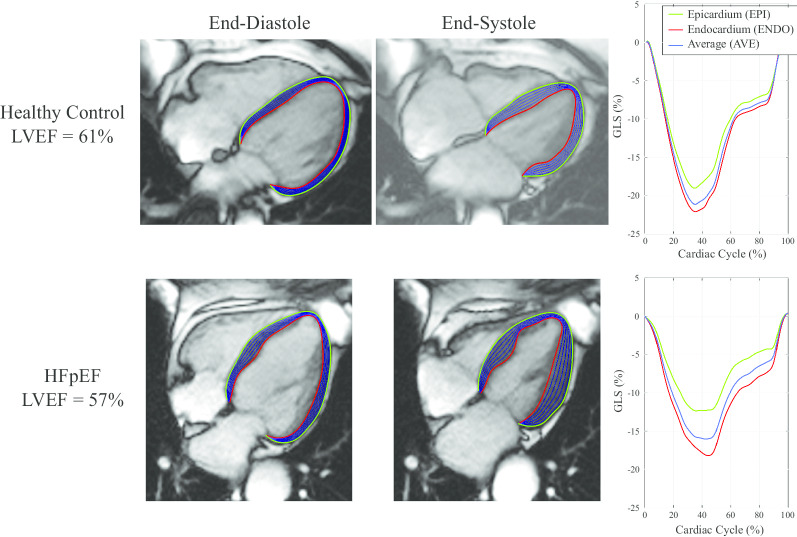
Layer-specific contours in long axis cine images, at endocardial (red), epicardial (green) and equally spaced intramyocardial contours (blue) at end-diastole and end-systole used for the calculation of layer-specific strain. A subset of the 20 total intramyocardial contours used for calculation of average strain are shown. Sample tracings for a four-chamber view are shown for a health control (top) and a patient with heart failure (HF) with preserved ejection fraction (HFpEF) (bottom). Strain values at each layer over the full cardiac cycle are shown on the right

**Fig. 3:**
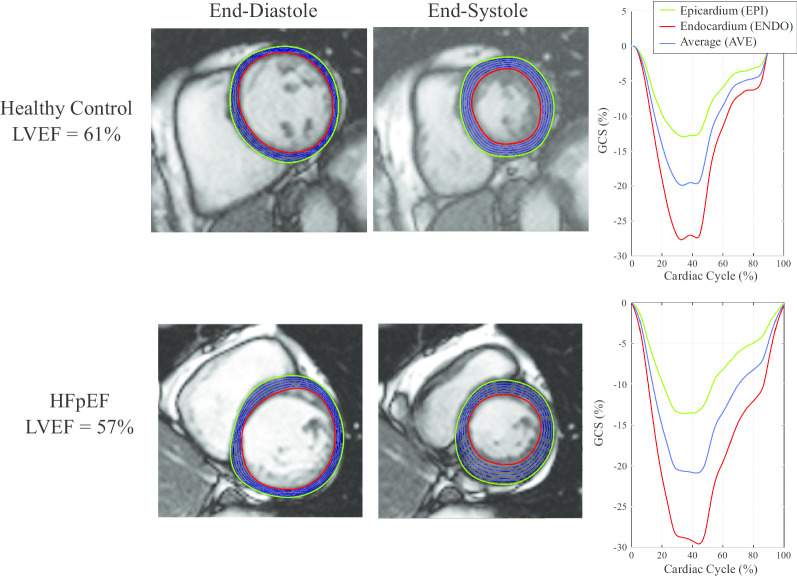
Layer-specific contours in short axis cine images, at endocardial (red), epicardial (green) and equally spaced intramyocardial contours (blue) at end-diastole and end-systole used for the calculation of layer-specific strain. A subset of the 20 total intramyocardial contours used for calculation of average strain are shown. Sample tracings for a mid-ventricular short-axis view are shown for a health control (top) and a patient with HFpEF (bottom). Strain values at each layer over the full cardiac cycle are shown on the right

Strain was measured from bSSFP short and long axis cine images using a feature tracking approach develop in-house based on b-spline non-rigid registration [24]. The strain evaluation procedure is summarized in Fig. 1. First, in-plane displacement fields were calculated from registration of all images in each cine image series to the reference end-diastolic image frame, separately for each short-axis and long-axis slice (Fig. 1a and b). Manual tracing was limited to tracing of endocardial (red) and epicardial (green) borders (Figs. 2 and 3) on the end-diastolic image frames (Fig. 1c). Tracings were completed by an experienced interpreter for all subjects (LX) who was blinded to clinical data and conventional CMR measures. Equally spaced contours between the endocardial and epicardial contours (blue) were generated at the end-diastolic frame (a subset of longitudinal contours are shown in Figs. 2 and 3 and 1d). A similar automated procedure was repeated for the radial direction. Subsequently, all end-diastolic contours were automatically propagated to all image frames over the full cardiac cycle using the previously calculated feature tracking displacement fields (Fig. 1d). Strain in each slice was calculated independently for all contours as the fractional change in length of the contour from end-diastole (L_0_) to end-systole (L) relative to end-diastolic length, reported as a percentage, (L − L_0_)/L_0_*100 (Lagrangian strain), as shown in Fig. 1d [25, 26]. GLS was calculated separately for the endocardium (GLS_ENDO), the epicardium (GLS_EPI) and the average of all contours (GLS_AVE). Peak systolic strain from all long axis slices were averaged to provide the reported global values (Fig. 2, right panels). The ratio, GLS_ENDO/GLS_EPI, as well as the absolute and relative differences between GLS_ENDO and GLS_EPI were also calculated for each subject. Similarly, global circumferential systolic strains (GCS_ENDO, GCS_EPI, GCS_AVE) were calculated as the average of the peak strains from two short-axis slices (mid and mid-basal) (Fig. 3). The ratio, GCS_ENDO/GCS_EPI, as well as the absolute and relative differences between GCS_ENDO and GCS_EPI were also calculated for each subject. Finally, global radial strains (GRS) were calculated separately from both long axis (GRS_LAX) and short axis slices (GRS_SAX). Contour lengths for the calculation of radial strains were measured as the distance between the endocardial and epicardial borders at end-diastole and end-systole (Fig. 1d). Radial strain for each slice was calculated as the average radial strain from all contours, and GRS as the average from all slices, for the two short-axis (GRS_SAX) and three long-axis slices (GRS_LAX), respectively.

GLS and GCS strains at all layers were also calculated using commercially available CMR feature tracking software (CVI42, Circle Cardiovascular Imaging) in a subset of 202 subjects, for a comparison of feature tracking methodology used in current study.

### Statistical approach

Continuous variables were expressed as mean ± standard deviation or median (25th, 75th percentile), as appropriate. Categorical variables were expressed as frequency and percentage. For missing data, the data was assumed to be missing at random. Our cohort had missing values for N-terminal prohormone of b-type natriuretic peptide (NT-proBNP, 11.0% in total, including 2.3% in healthy control, 3.5% in patients at risk for HF, 5.2% for patients with HF) and creatinine (8.6% % in total, including 3.1% in healthy control, 1.3% in patients at risk, 4.2% in HF patients). Multiple imputation by chained equations with 50 imputed data sets was used to generate missing data based on all candidate predictors and outcomes. We averaged results from the 50 imputations [27].

Two sample t-test (or Mann–Whitney U test) or one-way analysis of variances with Bonferroni post-hoc correction (or Dunn’s test) were used to compare continuous variables among groups of patients, as appropriate. Chi-square was used to compare the categorical baseline characteristics. The normal distribution of continuous variables was tested by the Shapiro–Wilk normality test. We applied logarithmic transformation to NT-proBNP and creatinine. Correlation between continuous variables was tested by Pearson correlation.

Clinical outcome was all-cause mortality over a 5-year follow-up. Univariable Cox proportional regression was performed for all demographic parameters, cardiovascular risk factors, cardiovascular disease history, concomitant diseases and CMR-derived imaging parameters. Sex and the parameters with univariable p value < 0.2, including age, systolic blood pressure, current smoker, presence of heart failure, coronary artery disease, atrial fibrillation or atrial flutter, chronic obstructive pulmonary disease, log (NT-proBNP) and log (creatinine) entered the forward selection approach based on Akaike Information Criterion (AIC) to build the optimal set of predictors as the base model for adjustment. In the multivariable Cox proportional hazard analysis conventional LV measurements, GRS, and layer-specific strains for GLS, GCS were each adjusted with the base model to test their independent association with the outcome. Additionally, the association of layer-specific strains and conventional LV measurements with clinical outcomes were quantified using the AIC values, where the lowest AIC score (AIC_minimum_) indicates the best outcomes model. Each compared model included the base model and a single evaluated CMR parameter. For comparison of AIC values (between CMR parameters), Δ_*i*_ = AIC_*i*_ – AIC_minimum_ is the difference between Model_*i*_ and best fit model. If Δ_*i*_ ≤ 2, there is substantial support for similar predictive performance of the best fit model and the i-th model; for 4 ≤ Δ_*i*_ ≤ 7 there is considerably less support for *i-*th model; for Δ_*i*_ > 10 there is no support for *i-*th model [28]. Finally, the incremental value of outcomes prediction by layer-specific strain over the other CMR variables was evaluated using the likelihood ratio test.

Intra-observer and inter-observer reproducibility of all strain parameters were evaluated by intra-class correlation coefficient and coefficient of variation in 20 randomly selected subjects with blinding to clinical data (the same subjects were used for both intra-observer and inter-observer calculations). Statistical analyses were performed using STATA (version 16.0, StataCorp LP, College Station, Texas, USA). A p-value less than 0.05 was considered significant for all tests.

## Results

### Demographics

From 466 subjects who underwent CMR exams, 453 subjects with analyzable images were identified: controls (n = 77), at-risk (n = 143), HFpEF (n = 87), HFmrEF (n = 88), and HFrEF (n = 58).

For statistical analysis of group differences, two subgroups of subjects were considered, those with preserved LVEF (> 55%), consisting of healthy controls, patients at-risk for HF and HFpEF (p-value 1 in Tables 1 and 2), and the three HF groups (p-value 2). Survival analysis included patients at-risk or with HF.

**Table 1:** Baseline Characteristics in the 5 Subgroups of Subjects

	Healthy controls (n = 70)	At risk for heart failure (n = 126)	HFpEF (n = 87)	p-value 1	HFmrEF (n = 88)	HFrEF (n = 58)	p-value 2
Preserved ejection fraction							
Age, year	59 (52,69)*^†^	65 (60,72)*	73 (64,81)	< 0.001	70 (60,75)^¶^	66 (59,77)^¶^	0.005
Male	29 (37.1%)	53 (42.1%)	34 (39.1%)	0.78	61 (69.3%)	40 (69.0%)	< 0.001
BMI, (kg/m^2^)	28 (24,30)*^†^	29 (25,34)*	30 (28,35)	< 0.001	29 (26,33)^¶^	29 (26,32)^¶^	0.013
Systolic blood pressure, (mmHg)	128 (116,140)^†^	137 (123,151)	130 (121,146)	0.070	125 (115,136)^¶^	124 (111,133)^¶^	0.006
Heart rate, bpm	67 (60,76)	68 (61,76)	64 (60,76)	0.16	64 (60,75)	68 (60,76)	0.30
NYHA Class			2.1 ± 0.7	N/A	1.9 ± 0.7	2.0 ± 0.7	0.30
Coronary artery disease	0	22 (17.5%)	37 (42.5%)	< 0.001	40 (45.5%)	29 (50%)	0.68
Hypertension	0	100 (79.4%)	75 (86.2%)	< 0.001	56 (63.6%)	37 (63.8%)	0.001
Atrial fibrillation/flutter	0	19 (15.1%)	34 (39.1%)	< 0.001	36 (40.9%)	23 (39.7%)	0.98
Chronic obstructive pulmonary disease	0	8 (6.4%)	18 (20.1%)	< 0.001	15 (17.1%)	12 (20.7%)	0.79
Chronic kidney disease	0	9 (7.1%)	22 (25.3%)	< 0.001	17 (19.3%)	11 (19.0%)	0.55
Beta blocker	0	34 (27.0%)	64 (73.6%)	< 0.001	79 (89.8%)^¶^	54 (93.1%)^¶^	0.001
ACEi or ARB	0	86 (68.3%)	73 (83.9%)	< 0.001	76 (83.6%)	50 (86.2%)	0.88
NT-proBNP (pmol/l)	5 (3,10)*^†^	7 (4,16)*	66 (19,132)	< 0.001	57 (24,147)	104 (52,253)^¶^^§^	0.003
Creatinine (mol/l)	78 (67,86)*	77 (67,89)*	96 (76,123)	< 0.001	97 (80,122)	93 (77,110)	0.49

*HFpEF* heart failure with preserved ejection fraction, *HFmrEF* heart failure with midrange ejection fraction, *HFrEF* heart failure with reduced ejection fraction, *BMI* body mass index, *NYHA* New York Heart Association Classification, *COPD* chronic obstructive pulmonary disease, *ACEI* Angiotensin converting enzyme inhibitor, *ARB* Angiotensin II receptor blocker, *NT-proBNP* N-terminal pro b-type natriuretic peptide

p-values 1 were derived from comparison among the three subgroups with preserved ejection fraction and p-values 2 for the three subgroups of patients with heart failure

*Significantly different from HFpEF in comparison among 3 subgroups with preserved LVEF

^†^Significantly different from patients at risk for heart failure in comparison among three subgroups with preserved LVEF

^¶^Significantly different from HFpEF in comparison among three subgroups with heart failure

^§^Significantly different from HFmrEF in comparison among three subgroups with heart failure

Continuous variables were expressed as mean ± standard deviation or median (25th, 75th percentile), as appropriate

**Table 2:** Cardiac structure and functions in the 5 subgroups of subjects

	Healthy controls (n = 70)	At risk for heart failure (n = 126)	HFpEF (n = 87)	p-value 1	HFmrEF (n = 88)	HFrEF (n = 58)	p-value 2
Preserved ejection fraction							
LVEF (%)	63 (61,67)^†^	66 (62,70)*	63 (59,68)	0.008	48 (44,52) ^¶^	32 (25,38)^¶^^§^	< 0.001
LVEDVI (ml/m^2^)	74 (68,83)	75 (65,87)	76 (68,86)	0.73	100 (81,114)^¶^	134 (100,165)^¶^^§^	< 0.001
LVESVI (ml/m^2^)	27 (23,31)	25 (21,31)	28 (22,33)	0.16	51 (40,62)^¶^	90 (64,121)^¶^^§^	< 0.001
LVM index, (g/m^2^)	55 (49,61)*^†^	61 (51,71)*	66 (56,79)	< 0.001	76 (63,88)^¶^	91 (74,110)^¶^^§^	< 0.001
LVM/LVEDV	0.72 (0.66,0.79)*^†^	0.80 (0.70,0.91)*	0.85 (0.73,0.98)	< 0.001	0.79 (0.66,0.88)^¶^	0.67 (0.60,0.76)^¶^^§^	< 0.001
RWT	0.31 (0.27,0.34) *	0.32 (0.28,0.37)*	0.34 (0.29,0.40)	0.019	0.32 (0.27,0.37)^¶^	0.28 (0.24,0.32)^¶^^§^	< 0.001
RVEF	58 (55,64)^†^	62 (57,67) *	60 (52,64)	0.021	52 (46,58)^¶^	49 (41,56)^¶^^§^	< 0.001
RVEDVI, (ml/m^2^)	74 (63,83)	75 (61,88)	71 (63,91)	0.74	81 (67,97)^¶^	79 (68,108)^¶^	0.070
RVESVI, (ml/m^2^)	30 (23,35)	28 (22,35)	31 (24,39)	0.27	38 (28,48)^¶^	39 (31,57)^¶^	< 0.001
GLS_EPI (%)	− 16.5 ± 2.4*^†^	− 15.5 ± 2.7*	− 14.1 ± 3.0	< 0.001	− 11.6 ± 2.1^¶^	− 8.2 ± 2.2^¶^^§^	< 0.001
GLS_AVE (%	− 19.6 ± 2.5*	− 19.2 ± 3.1*	− 17.9 ± 3.3	< 0.001	− 14.0 ± 2.3^¶^	− 9.4 ± 2.7^¶^^§^	< 0.001
GLS_ENDO (%)	− 21.1 ± 2.6	− 21.2 ± 3.4	− 20.1 ± 3.7	0.050	− 15.3 ± 2.9^¶^	− 10.0 ± 3.2^¶^^§^	< 0.001
GLS_ENDO/GLS_EPI	1.3 (1.2,1.4)*^†^	1.4 (1.3,1.5)*	1.4 (1.3,1.5)	< 0.001	1.3 (1.2,1.4)^¶^	1.2 (1.2,1.3)^¶^^§^	< 0.001
Absolute GLS layer difference, (%)	− 4.7 (− 5.8, − 3.3)*^†^	− 5.7 (− 7.4, − 4.1)	− 5.6 (− 7.5, − 4.3)	< 0.001	− 3.5 (− 5.1, − 2.3)^¶^^§^	− 2.1 (− 2.7, − 1.0)^¶^^§^	< 0.001
Relative GLS layer difference (%)	21.9 (16.5,27.0)*^†^	27.3 (20.4,32.5)*	28.9 (23.4,34.4)	< 0.001	25.4 (16.1,29.9)^¶^^§^	17.9 (13.4,25.8)^¶^^§^	< 0.001
GCS_EPI (%)	− 11.1 ± 2.8	− 10.2 ± 2.5	− 10.2 ± 3.4	0.072	− 7.8 ± 2.4^¶^	− 5.7 ± 2.2^¶^^§^	< 0.001
GCS_AVE (%)	− 19.9 ± 3.4	− 19.9 ± 3.3	− 19.3 ± 4.0	0.40	− 13.9 ± 3.1^¶^	− 9.3 ± 3.1^¶^^§^	< 0.001
GCS_ENDO (%)	− 29.9 ± 4.7	− 31.1 ± 5.3	− 29.9 ± 6.1	0.17	− 21.1 ± 4.9^¶^	− 13.1 ± 4.6^¶^^§^	< 0.001
GCS_ENDO/GCS_EPI	2.6 (2.4,3.1)*^†^	3.0 (2.6,3.6)	2.9 (2.4,4.0)	0.004	2.6 (2.3,3.3)^¶^	2.3 (1.9,2.9)^¶^^§^	< 0.001
Absolute GCS layer difference (%)	− 18.8 (− 21.1, − 16.3)^†^	− 20.8 (− 24.3, − 17.8)*	− 19.0 (− 23.1, − 16.1)	0.002	− 13.0 (− 16.2, − 9.8)^¶^	− 7.5 (− 8.7, − 5.1)^¶^^§^	< 0.001
Relative GCS layer difference (%)	61.8 (58.4,67.5) *^†^	67.0 (62.1,72.1)	65.4 (57.8,74.8)	0.004	62.0 (57.1,69.2)^¶^	56.8 (47.4,64.9)^¶^^§^	< 0.001
GRS_LAx (%)	48.9 ± 10.7*	47.5 ± 12.8*	41.1 ± 13.8	< 0.001	28.5 ± 9.4^¶^	18.7 ± 7.7^¶^^§^	< 0.001
GRS_SAx (%)	46.7 ± 12.4*	46.3 ± 15.1*	38.6 ± 16.7	< 0.001	27.0 ± 9.0^¶^	17.7 ± 8.1^¶^^§^	< 0.001

*HFpEF* heart failure with preserved ejection fraction; *HFmrEF* heart failure with midrange ejection fraction; *HFrEF* heart failure with reduced ejection fraction; *LVEF* left ventricular ejection fraction; *LVEDVI* left (right) ventricular end-diastolic volume indexed to ideal body surface area; *LVESVI* left (right) ventricular end-systolic volume indexed to ideal body surface area; *LVM* left ventricular mass; *RVEF* right ventricular ejection fraction, *RVEDVI* right ventricular end-diastolic volume index; *RVESVI* right ventricular end-systolic volume index, *RWT* relative wall thickness; *GLS_EPI *epicardial global longitudinal strain_;_ *GLS_AVE *average global longitudinal strain_;_ *GLS_ENDO* endocardial global longitudinal strain_;_ *GCS_EPI *picardial global circumferential strain_;_ *GCS_AVE* average global circumferential strain_;_ *GCS_ENDO* endocardial global circumferential strain; *GRS* global radial strain_;_ *LAx* long axis; *SAx* short axis

Absolute strain layer difference = endocardial strain—epicardial strain; relative strain layer difference = (endocardial strain—epicardial strain)/endocardial strain

p-values 1 were derived from comparison among the three subgroups with preserved ejection fraction and p-value 2 for the three subgroups of patients with heart failure

*Significantly different from HFpEF in comparison among three subgroups with preserved LVEF

^†^Significantly different from patients at risk for heart failure in comparison among three subgroups with preserved LVEF

^¶^Significantly different from HFpEF in comparison among three subgroups with heart failure

^§^Significantly different from HFmrEF in comparison among three subgroups with heart failure

Continuous variables were expressed as mean ± standard deviation or median (25th, 75th percentile), as appropriate

Those with HFpEF were older and had higher body mass index, higher medication use, more concomitant disease and higher serum NT-proBNP, as compared to healthy controls and those at-risk (all ps < 0.001, Table 1). Among the three HF groups, all had similar serum creatinine and concomitant disease, however those with HFpEF were slightly older with a larger proportion of females, slightly higher body mass index, and lower rate of beta blocker use (all ps < 0.05) and those with HFrEF had higher level of serum NT-proBNP with lower systolic blood pressure (both ps < 0.05) (Table 1).

### Ventricular structure and function

Within the HF groups, there was significantly lower LVEF, lower strains and larger LVM and volumes from HFpEF to HFmrEF and to HFrEF groups, respectively (Table 2, all ps < 0.001). LVEF was similar within the preserved LVEF groups (controls, at-risk and HFpEF) by definition. All circumferential strain components, GLS_ENDO as well as LV and RV volumes were also similar in the preserved LVEF groups. GLS_EPI was the only strain parameter that distinguished all three groups in post-hoc analysis, with incrementally reduced systolic function from healthy controls, to those at-risk to HFpEF (− 16.5 ± 2.4% vs. − 15.5 ± 2.7% vs. − 14.1 ± 3.0%, p < 0.001). Other parameters, including GLS_AVE and GRS, also identified systolic dysfunction in the HFpEF group, with significantly reduced values versus healthy controls and at-risk groups (Table 2). Finally, HFpEF patients were also distinguished from healthy controls by significantly increased differences in endocardial and epicardial strains, measured as absolute or relative differences or a ratio (ENDO/EPI), for both GLS and GCS. Both LVM index and LVM/LVEDV also distinguished all three groups in post-hoc analysis, with a significant stepwise increase from controls, to those at-risk to HFpEF.

**Fig. 4:**
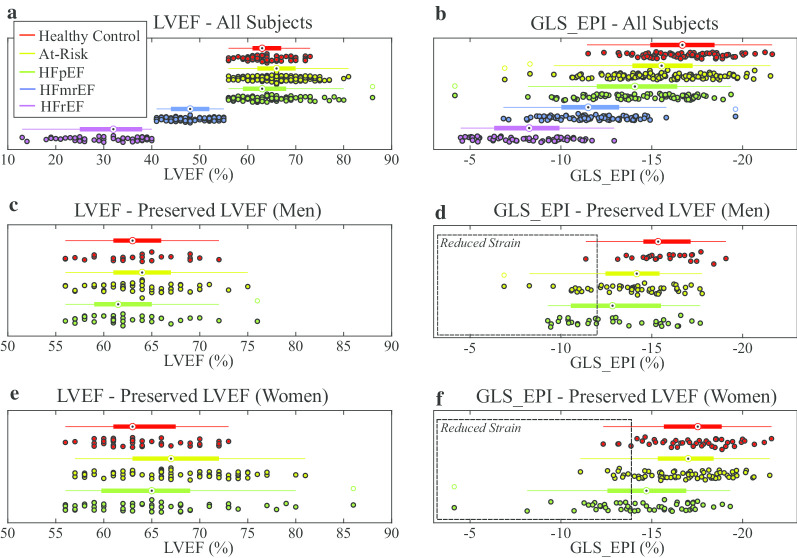
Scatter and box plots of LV ejection fraction (LVEF) (**a**) and epicardial global longitudinal strain (GLS_EPI) (**b**) in all five groups. For each group, box plots show the median value, 25th and 75th percentiles and the full extent of the data. Outliers are identified as unfilled circles in the box plot. Scatter and box plots for LVEF (**c** and **e**) and GLS_EPI (**d** and **f**) in the preserved LVEF groups are shown with grouping by sex. Individuals with reduced GLS_EPI values are contained within the dashed boxes in **d** (men: 11% of controls, 17% of the at risk group and 32% of HFpEF are within the box) and **f** (women: 9% of healthy controls, 14% of the at risk group and 45% of HFpEF are within the box). All subjects in the preserved LVEF groups have normal LVEF by definition

**Fig. 5:**
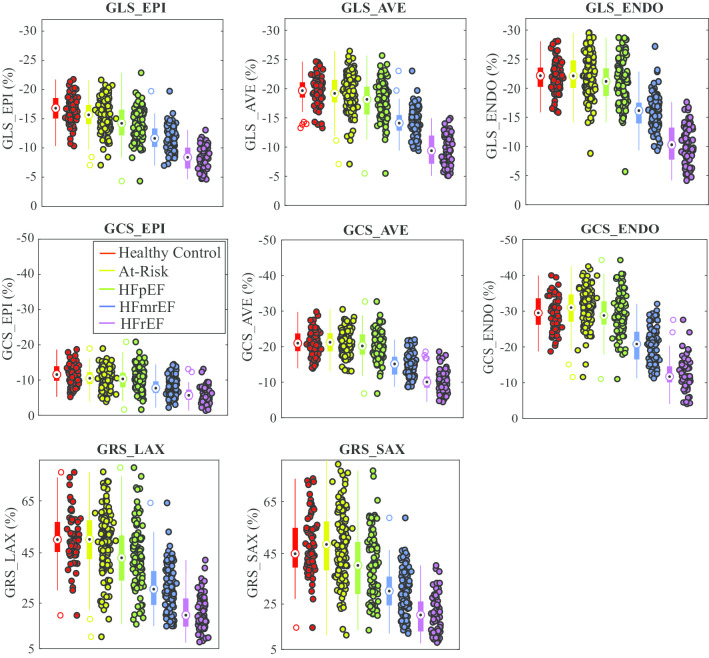
Scatter and box plots for LVEF and all strain components for all five groups. Box plots show the median value, 25th and 75th percentiles and the full extent of the data. Outliers are identified as unfilled circles in the box plot

LVEF values were strongly overlapping in the three preserved LVEF groups and distinct in the three HF groups, by definition, as illustrated in Fig. 4a for all study subjects. GLS_EPI values, conversely, had overlapping values in all HF patients while also being reduced in the HFpEF group versus the other LVEF-matched groups (Fig. 4b). Using the 90th percentile in the control group as a cutoff for identification of systolic dysfunction, with consideration of sex differences, GLS_EPI identified 35/87 HFpEF patients (GLS_EPI > − 11.4% cutoff in males (Fig. 4d), GLS_EPI > -14.2% cutoff in females (Fig. 4f)), while GLS_ENDO identified a much smaller subgroup of 18/87 with reduced function (GLS_ENDO >− 16.0% cutoff in males, GLS_ENDO > − 18.7% cutoff in females) (not shown). Similar scatter plots including all study subjects are shown for all strain components (Fig. 5). All three long axis and two short axis slices were included in the strain analysis for all subjects.

GLS and GCS values were significantly reduced from ENDO to EPI layers in all groups. Additionally, the ratio of strains between the layers and the absolute and relative strain differences between the layers illustrate the relatively lower strains on the EPI (Table 2). Within the HF groups, the differences between ENDO and EPI strains (ratio, absolute and relative differences) were significantly reduced from HFpEF to HFmrEF to HFrEF (Table 2).

**Fig. 6:**
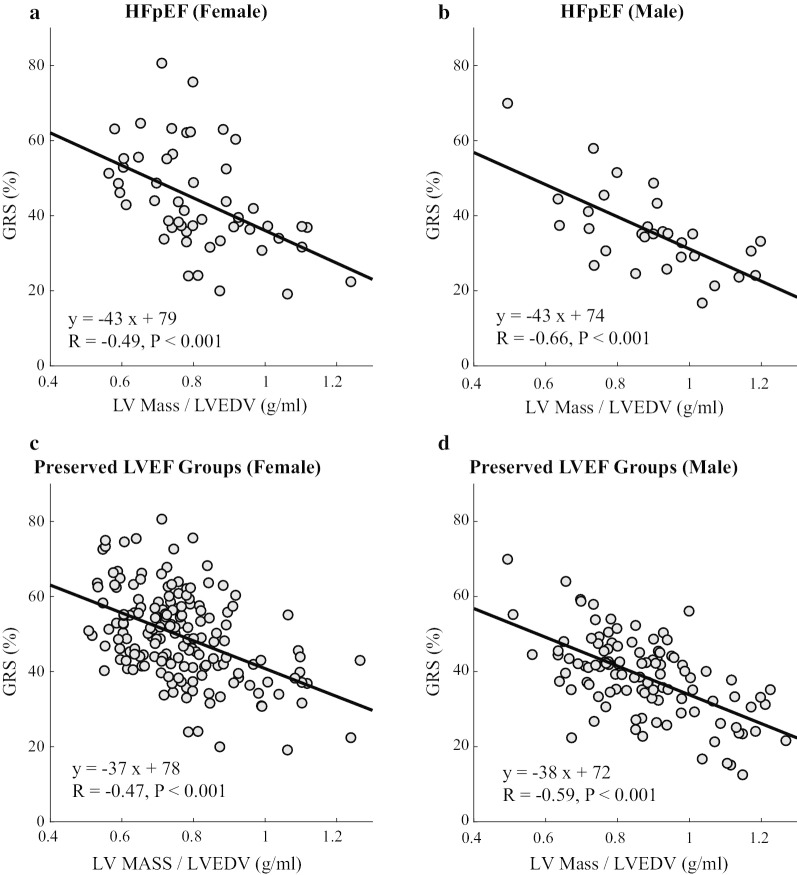
Relationship between GRS and LV Mass / LVEDV (concentricity) in HFpEF patients (**a** and **b**) and in all subjects with preserved LVEF (**c** and **d**). See Table 2 for abbreviations

The significant linear relationship between strain and LV structure in the preserved LVEF groups is illustrated by comparison of GRS (GRS_SAX) and LVM/LVEDV (concentricity) values in HFpEF (top row) and all individuals with preserved LVEF (bottom row), with separate results by sex (Fig. 6). Similar significant linear correlations were observed when comparing GLS_EPI and LVM/LVEDV (not shown). However, all GCS components had no significant relationship with LVM/LVEDV (not shown).

### Layer-specific strain in healthy controls

Among the 77 healthy controls subjects (29 male, median age 59 years), male subjects had lower strains than females at all locations except for GCS_ENDO which were similar (Table 3). For both GLS and GCS, values were lowest at the epicardium with a stepwise increase to the average value in myocardium and to the endocardium (all ps < 0.001). The magnitude of GRS values measured from long axis (GRS_LAX) and short axis slices (GRS_SAX) were similar (p > 0.05).

**Table 3:** Comparison of layer-specific strain, LV mass and relative wall thickness between two sexes in healthy controls

	Male (n = 29)	Female (n = 48)	p-value
LVEF (%)	62 (59,65)	63 (60,67)	0.50
GLS_EPI (%)	− 15.0 ± 2.4	− 17.1 ± 2.3	< 0.001
GLS_AVE (%)	− 18.7 ± 2.6	− 20.5 ± 2.5	0.006
GLS_ENDO (%)	− 19.6 ± 2.6	− 21.4 ± 2.7	0.005
GLS_ENDO/GLS_EPI	1.32 ± 0.12	1.26 ± 0.11	0.035
Absolute GLS layer difference, %	− 5.0 (− 5.6, − 3.8)	− 4.2 (− 5.6, − 3.2)	0.33
Relative GLS layer difference, %	24.2 (20.1,28.0)	19.7 (15.6,25.7)	0.030
GCS_EPI (%)	− 9.6 ± 2.6	− 11.6 ± 2.7	0.002
GCS_AVE (%)	− 18.5 ± 3.3	− 20.1 ± 3.4	0.051
GCS_ENDO (%)	− 28.9 ± 4.7	− 29.7 ± 4.9	0.50
GCS_ENDO/GCS_EPI	2.97 (2.61,3.47)	2.56 (2.32,2.86)	0.001
Absolute GCS layer difference (%)	− 18.7 (− 21.7, − 16.6)	− 18.3 (− 20.4, − 15.8)	0.29
Relative GCS layer difference (%)	66.3 (61.6,71.2)	61.0 (56.9,65.1)	0.001
GRS_LAx (%)	43.6 ± 10.6	50.6 ± 10.3	0.006
GRS_SAx (%)	41.7 ± 11.7	49.1 ± 12.2	0.011
LVM index,g/m^2^	60 (57,71)	52 (47,57)	0.005
LVM/LVEDV	0.81 (0.70,0.93)	0.74 (0.64,0.83)	< 0.001
RWT	0.34 (0.30,0.37)	0.30 (0.26,0.32)	< 0.001

Continuous variables were expressed as mean ± standard deviation or median (25th, 75th percentile), as appropriate

p-values were derived from comparison between two sexes

### Clinical outcomes

During a 5-year follow-up for the at-risk and HF subgroups, there were 33 events of all-cause mortality. The base model included age and log (NT-proBNP) for multivariable analysis. After adjustment with the base model, GLS_AVE and GRS (for both LAX and SAX-derived GRS) were the only independent predictors of mortality, with adjusted hazard ratio 1.10, 1.03 and 1.03, respectively, for 1% absolute decrease of strains, all ps < 0.05. LVEF and GLS_ENDO were unable to independently predict outcome (Table 4). Additionally, AIC values for the layer-specific GLSs, GCSs and conventional LV measurements were compared to evaluate the relative strength of their association with clinical outcomes. GLS_AVE and GRS had the lowest AIC values, which demonstrates the strongest association with the clinical outcome (Table 5). For the remaining parameters, GLS_ENDO, GLS_EPI, all GCS components and conventional LV measures (LVEF, indexed LVEDV (LVEDVI), indexed LVESV (LVESVI) and indexed LVM) had considerably less support for similar performance as GLS_AVE and GRS for outcomes prediction_,_ with ΔAIC values of 3.5 to 6.1 [28]. The use of GLS_AVE had significant incremental value for prediction of outcomes over LVEF and GLS_ENDO (Δχ^2^ = 5.1 and 7.4, respectively, both ps < 0.05), by the likelihood ratio test. GRS, from both at short axis and long axis images, had incremental value over LVEF (Δχ^2^ = 4.4 and 4.8, respectively, both ps < 0.05).

**Table 4:** Cox proportional hazard regression analysis for the outcome of 5-year all-cause mortality

	Patients with HF or at risk for HF- 376 subjects (33 events)				
	Univariable analysis			Multivariable analysis	
	HR (95% CI)	p-value	Model χ^2^	HR (95% CI)	p-value
Age at CMR, per 10 year increase	2.37 (1.58,3.56)	< 0.001	21.5		
Male gender	0.66 (0.32,1.34)	0.25	1.4		
BMI, per 1 kg/m^2^ increase	1.00 (0.94,1.06)	0.99	0.1		
Systolic blood pressure, per 10 mmHg increase	0.80 (0.66,0.98)	0.031	0.2		
Current smoker	1.34 (0.93,1.91)	0.12	2.6		
History of heart failure	6.43 (1.96,21.08)	0.002	15.3		
Hypertension	1.07 (0.48,2.38)	0.86	0.1		
Coronary artery disease	2.15 (1.11,4.17)	0.023	5.1		
Atrial fibrillation/flutter	2.81 (1.42,5.58)	0.003	8.6		
Diabetes	0.81 (0.39,1.70)	0.58	0.3		
COPD	1.92 (0.87,4.26)	0.11	2.3		
Chronic kidney disease	1.20 (0.50,2.91)	0.69	0.2		
Log (NT-proBNP), per 1 unit increase	1.84 (1.44,2.36)	< 0.001	27.5		
Log (Creatinine), per 1 unit increase	3.20 (1.37,7.49)	0.007	6.2		
LVEF, per 10% decrease	1.37 (1.10,1.71)	0.004	7.6	1.22 (0.95,1.58)	0.12
LVEDVI, per 10 ml/m^2^ increase	1.09 (1.00,1.18)	0.043	3.5	1.06 (0.97,1.15)	0.19
LVESVI, per 10 ml/m^2^ increase	1.11 (1.02,1.20)	0.013	5.0	1.07 (0.97,1.17)	0.16
LVM index, per 10 g/m^2^ increase	1.14 (1.00,1.29)	0.051	3.3	1.09 (0.95,1.25)	0.25
LVM/LVEDV, per 0.1 increase	0.98 (0.82,1.17)	0.82	0.1	0.92 (0.81,1.03)	0.14
GLS_EPI, per 1% absolute decrease	1.11 (1.04,1.18)	0.002	9.4	1.09 (0.98,1.22)	0.11
GLS_AVE, per 1% absolute decrease	1.14 (1.06,1.23)	< 0.001	12.5	1.10 (1.01,1.20)	0.023
GLS_ENDO, per 1% absolute decrease	1.10 (1.04, 1.18)	0.002	9.2 LR chi2 (2) = 1 LR chi2 (2) = 1	1.06 (0.98, 1.15)	0.15
GCS_EPI, per 1% absolute decrease	1.01 (0.91,1.13)	0.83	0.1	0.99 (0.90,1.10)	0.87
GCS_AVE, per 1% absolute decrease	1.05 (0.99, 1.12)	0.10	2.6	1.02 (0.95,1.09)	0.57
GCS_ENDO, per 1% absolute decrease	1.04 (1.00,1.08)	0.044	4.0	1.02 (0.98,1.06)	0.41
GRS_LAx, per 1% decrease	1.03 (1.01,1.05)	0.001	12.2	1.03 (1.00,1.05)	0.024
GRS_SAx, per 1% decrease	1.03 (1.01,1.05)	0.002	11.9	1.03 (1.00,1.05)	0.019

Base model: age + log (NT-proBNP)

See Tables 1, 2 for abbreviations

**Table 5:** Discrimination performance of layer-specific Strains by Akaike information Criterion (AIC) for death at 5 years

	AIC	ΔAIC
BM + LVEF	355.7	3.7
BM + LVEDVI	356.5	4.5
BM + LVESVI	356.4	4.4
BM + LVM indexi	356.9	4.9
BM + GLS_EPI	355.5	3.5
BM + GLS_AVE	352.8	0.8
BM + GLS_ENDO	355.7	3.7
BM + GCS_EPI	358.1	6.1
BM + GCS_AVE	357.5	5.5
BM + GCS_ENDO	357.4	5.4
BM + GRS_LAx	352.3	0.3
BM + GRS_SAx	352.0	0

*BM* base model, including age and log (NT-proBNP).

ΔAIC = AIC_*i*_*—*AIC_*minimum*_ (AIC_*minimum*_ = AIC_GRS_SAX_). The model with lowest AIC score (AIC_minimum_) indicates the best model. See Table 2 for abbreviations.

### Reproducibility

The intra-observer reproducibility of all layer-specific strains was excellent; CoV values ranged from 2.7 to 5.4%, with 4.3% for GLS_EPI and 2.7% for GLS_ENDO, and ICC values ranging from 0.97 to 0.99, with 0.98 for GLS_EPI and 0.99 for GLS_ENDO. The inter-observer reproducibility of all layer-specific strains was also excellent; CoV values ranged from 4.9 to 9%, with 4.9% for GLS_EPI and 8.7% for GLS_ENDO, and ICC ranged from 0.92 to 0.98, with 0.92 for GLS_EPI and 0.98 for GLS_ENDO. Comparison of the custom feature tracking software used in the current study and the commercially available CVI42 for all GLS and GCS components in 202 subjects illustrated good agreement between the methods. (Additional file 1: Fig. 1).

## Discussion

The main findings of the current study are that consideration of measurement layer for global strain is necessary for optimal identification of dysfunction and outcomes prediction in HF. The endocardium-specific strains were shown to have poorest performance both for detection of systolic dysfunction and outcomes prediction.

### Layer-dependence of systolic dysfunction and association with remodeling in HFpEF

In subjects with preserved LVEF, GLS_EPI distinguished all three groups with preserved LVEF and identified ~ 40% of HFpEF patients as having reduced systolic function. In contrast, GCS components at all layers and GLS_ENDO performed similarly to LVEF (i.e. values were comparable in healthy controls, those with HF risk factors and HFpEF). Similar to recent studies in healthy subjects [10, 29, 30] and in HF [19], GLS and GCS values were incrementally decreased from ENDO to EPI in all groups. In the current study, it was also found that the difference between ENDO and EPI strains in individuals (relative and absolute differences) were significantly higher in HFpEF as compared to healthy controls. This is in agreement with the patterns of preserved ENDO strains and reduced GLS_EPI in this HF group.

Additionally, indexed LVM and LVM/LVEDV were significantly increased in the HFpEF group as compared to at-risk and healthy controls. Together, these findings describe a HFpEF phenotype with preserved ENDO function, paralleling LVEF, but with increased strain reduction across the wall, potentially associated with increased LVM and concentricity (mass/volume). GRS was also significantly reduced in the HFpEF group as compared to healthy controls, and was shown to be associated with increased concentricity. A similar relationship was observed for GLS_EPI, with more impaired function in those with increased concentricity. These observed associations between strains and structure also exist in comparison of the healthy men and women, where men had larger LVM, larger LVM/LVEDV, lower GLS_EPI, lower GRS and larger differences between ENDO and EPI strains.

In the current study, the use of a higher cutoff of 55% for preserved LVEF [21, 22], as compared to the more commonly used 50%, was used to reflect the larger number of HF patients in the 50%-55% range but very small proportion of at-risk or control subjects in this range. He et al. showed that mildly decreased LVEF, in the range of 40–55% (matching our HFmrEF group in the current study), is associated with eccentric remodeling and decreased chamber contractility, from invasive pressure–volume analysis, most comparable to subjects with overt systolic HF. The use of the lower 50% cutoff for preserved LVEF would have resulted in a significantly lower LVEF in our HFpEF group, compared to at-risk and healthy controls, and a large number of individuals in the HFpEF that are outside of the normal range of LVEF values from the control group and literature values.

For many of the reported structural and functional parameters that distinguish HFpEF from healthy controls, the values in the at-risk group were intermediate, between the control and HFpEF groups, with significant group differences on post-hoc analysis, suggesting early changes in these parameters may contribute to or be associated with future development of HF.

When comparing the three HF groups alone (preserved, mid-range and reduced LVEF), all CMR parameters were significantly different between all groups, with larger volumes, increased LV mass and reduced strain (all components) from HFpEF to HFrEF. The differences between endocardial and epicardial strains also followed this pattern, with reduced differences between the layers from HFpEF to HFrEF, paralleling the reduction in concentricity (mass/volume).

### Heart failure outcomes

Among all strain components, only GLS_AVE and GRS were significantly predictive of mortality in HF patients and those at risk when including the key factors of age and NT-proBNP in the outcomes model. Both parameters had superior outcomes prediction performance as compared to commonly reported GLS_ENDO [6, 7, 11, 12, 31]. Not surprisingly, LVEF was also not predictive of outcomes, in agreement with large studies showing that HF outcomes are independent of LVEF, being similar for HFpEF, HFmrEF and HFrEF groups [4]. Similar to our findings, a recent study of 463 patients with HFpEF showed no association between endocardial GLS and mortality or a composite of mortality or rehospitalization at 1 year [32]. While reduced GLS_ENDO has previously been shown to have incremental value over LVEF in outcomes prediction in acute HF [7, 31], NT-proBNP, a robust and widely available predictor of mortality [33, 34], was not included in the reported outcomes models. Case in point, in the current study, GLS_ENDO, GLS_AVE and GLS_EPI and even LVEF were all significantly predictive of outcomes in univariable analysis, but of these only GLS_AVE remained independently associated with clinical outcomes when including NT-proBNP and age in the outcomes model. Similar to our findings, a recent CMR feature tracking study showed that GLS_AVE and GRS were predictive of outcomes in HF [35]. Different from our study, GCS_AVE was also shown to be predictive of outcomes. This may reflect differences in the patient cohorts and the overall limited number of outcomes. However, the authors acknowledged that lack of NT-pro-BNP in their study, for multivariable analysis of risk, was a limitation given the very strong predictive power of NT-pro-BNP in heart failure.

The limited improvement in endocardial-specific strain over LVEF for detection of dysfunction or prognosis might be expected given the strong relationship between volumetric function, from which LVEF is defined, and deformations of the endocardial surface. Recently, Stokke et al. highlighted the direct relationship between endocardial strains and LVEF, and that LV wall thickness becomes a modulator of this relationship when considering average strains, measured across the wall thickness [36]. Specifically, their mathematical model showed how thicker ventricles can have reduced average strains across the wall thickness in the presence of preserved LVEF. Similarly, MacIver et al. illustrated the mechanism by which mid-wall global strains can be reduced in the presence of normal LVEF as a result of increased wall thickness, based solely on geometry [37]. Similar geometric models linking wall thickness, LVEF and strains, with similar conclusions, have previously been described [38]. It is thus possible that the superior prognostic performance of GLS_AVE over endocardial strains reflects its dependence on both systolic strain and LV mass, where LV mass itself is predictive of cardiovascular outcomes [39, 40]. GRS had similar good outcomes prediction performance as compared to GLS_AVE. Like GLS_AVE, GRS directly incorporates geometric information from the full thickness of the myocardium. Additionally, reduced GRS was shown to be associated with increased concentricity (LV mass/LVEDV), so like GLS_AVE, may integrate structural remodeling and reduced function. GRS measured from short axis (GRS_SAX) or long axis (GRS_LAX) images had similar values in all groups and similar outcomes prediction performance. It is unclear why GLS_EPI was not significantly predictive of outcomes, given its sensitivity to systolic dysfunction in HF in the current study, and its association with structural remodeling, similar to GRS. It is possible that increased measurement variability at the epicardium has contributed to this finding. Similar to previous studies, reproducibility of ENDO strain was superior to those measured on the EPI [41].

It should be acknowledged that the conventional strain components, longitudinally or circumferentially, are not along the direction of fiber shortening. Fibers are helically orientated at the ENDO and EPI [8]. It is possible that reduced strain along a single direction at the EPI, for example, could reflect changes in fiber orientations and function across the thickness of the heart wall [42].

### Dependence on sex

Similar to previous studies [9, 30], men were shown to have lower absolute strain values than women for most strain components, necessitating the definition of sex-specific normal values.

### Study limitations

Our study has a modest sample size and number of events for outcomes analysis, a reflection of the clinical stability of our HF cohort, which may limit the generalizability of the reported findings. However, based on the suggested 10 outcome events per predictor, our use of age, log (NT-proBNP) with each CMR parameter separately (i.e. 3 predictors in the composite model), our observed 33 events (mortality at 5 years) are sufficient. The base model, while only including age and NT-proBNP was established based on AIC forward selection including all cardiovascular disease risk factors and disease history with p-values < 0.2 and is thus robust and representative. Also, age and NT-proBNP have previously been reported to be the strongest predictors of mortality among clinical characteristics in patients with HF or without HF [34]. An additional limitation is the generalizability of the reported findings given the heterogeneity of the underlying pathologies of HF, some of which may not follow the trends reported in the current study. For example, endocardial strains have been shown to have superior prognostic performance in ischemic disease [14, 15]. Nonetheless, the overall significant findings for GLS_EPI, GLS_AVE and GRS speak to the robustness of these metrics across a wide spectrum of functional abnormalities. The calculation of average strain across the wall is limited by the relatively poor contrast within the myocardium, which may lead to errors in this strain component. This is an intrinsic limitation of CMR feature tracking with conventional cine imaging, for which the myocardium has relatively uniform signal intensity. Two short axis slices at mid and mid-basal locations were used for assessment of circumferential and radial strains which would not be sensitive to apical wall motion abnormalities. The HFpEF group in the current study was slightly older than the healthy control and at-risk groups, which may contribute to the lower observed strains in this group, however, the relatively small expected decline in strains beyond 50 years of age [29] suggests these effects will be negligible. Age was also included as a co-factor in all statistical analyses to address potential age effects. CMR has lower availability than echocardiography for the measurement of strain. However, recent speckle tracking study of healthy subjects reported similar GLS layer-specific values to those reported in the controls in the current study [29, 30], suggesting that current echocardiographic methods are similar to CMR for layer specific stain evaluation. Also, direct comparison of strain measured with CMR feature tracking and speckle tracking echocardiography has shown good inter-technique agreement [43]. Finally, the reported findings remain to be verified in an independent validation cohort.

## Conclusion

Global strains measured on ENDO, EPI or average across the wall thickness are not equivalent for the identification of dysfunction or outcomes prediction in HF. The endocardium-specific strains were shown to have poorest all-around performance. GLS_AVE and GRS were the only CMR parameters to be significantly associated with 5-year all-cause mortality in multivariable analysis. GLS_EPI, GLS_AVE, GRS and the relative difference in endocardial and epicardial strains differentiated HFpEF patients from healthy controls, and increased LVM and LVM/LVEDV were generally associated with reduced strain in those with preserved LVEF.

## Supplementary information

**Additional file 1: Fig. 1**. Comparison of feature tracking software (custom software used in the current study versus CVI42) for calculation of layer-specific longitudinal and circumferential strains in a subset of 202 subjects (41 controls, 73 at-risk and 88 heart failure). See Table 2 for abbreviations.

## Data Availability

The data used in this study are available from the corresponding author on reasonable request.

## Abbreviations

ACC: American College of Cardiology
ACEI: Angiotensin converting enzyme inhibitor
AF/AFl: Atrial fibrillation/flutter
AHA: American Heart Association
AIC: Akaike information criterion
ARB: Angiotensin II receptor blocker
AVE: Average
BMI: Body mass index
BSA: Body surface area
bSSFP: Balanced steady state free precession
CMR: Cardiovascular magnetic resonance
ENDO: Endocardium/endocardial
EPI: Epicardium/epicardial
GCS: Global circumferential strain
GLS: Global longitudinal strain
GCS_AVE: Average global circumferential strain
GCS_ENDO: Endocardial global circumferential strain
GCS_EPI: Epicardial global circumferential strain
GLS_AVE: Average global longitudinal strain
GLS_ENDO: Endocardial global longitudinal strain
GLS_EPI: Epicardial global longitudinal strain
GRS: Global radial strain
HF: Heart failure
HFmrEF: Heart failure with midrange ejection fraction
HFpEF: Heart failure with preserved ejection fraction
HFrEF: Heart failure with reduced ejection fraction
LAx: Long axis
LV: Left ventricle/left ventricular
LVEDV: Left ventricular end-diastolic volume
LVEDVI: Left ventricular end diastolic volume index
LVEF: Left ventricular ejection fraction
LVESV: Left ventricular end-systolic volume
LVESVI: Left ventricular end-systolic volume index
LVM: Left ventricular mass
NT-proBNP: N-terminal pro b-type natriuretic peptide
NYHA: New York Heart Association Classification
RV: Right ventricle/right ventricular
RVEDV: Right ventricular end-diastolic volume
RVEDVI: Right ventricular end-diastolic volume index
RVESV: Right ventricular end-systolic volume
RVESVI: Right ventricular end-systolic volume index
RWT: Relative wall thickness
SAx: Short axis
TE: Echo time
TR: Repetition time
